# Hyperoside Stabilizes Redox–Mitochondrial–Inflammatory Networks in a Haloperidol-Induced Tardive Dyskinesia–Like Model

**DOI:** 10.3390/life16050814

**Published:** 2026-05-13

**Authors:** Hsiang-Chien Tseng, Mao-Hsien Wang, Kuo-Chi Chang, Chih-Pei Hsu

**Affiliations:** 1 Department of Anesthesiology, Shin Kong Wu Ho-Su Memorial Hospital, Taipei 11101, Taiwan; 2 School of Medicine, Fu Jen Catholic University, New Taipei City 24205, Taiwan; 3 Department of Anesthesia, En Chu Kon Hospital, New Taipei City 23702, Taiwan; 4 Institute of Taiwan Instrument Research, National Applied Research Laboratories, Hsinchu 300092, Taiwan; 5 Department of Chemical Engineering and Biotechnology, National Taipei University of Technology, Taipei 10608, Taiwan; 6 Division of Neurosurgery, Department of Surgery, Hsinchu Br. of Mackay Memorial Hospital, Hsinchu City 30071, Taiwan; 7 Department of Nursing, Mackay Junior College of Medicine, Nursing and Management, Taipei 11260, Taiwan; 8 Department of Medicine, Mackay Medical College, New Taipei City 252, Taiwan

**Keywords:** hyperoside, mitochondria, neuroinflammation, Nrf2, oxidative stress, tardive dyskinesia

## Abstract

Tardive dyskinesia (TD) is a persistent hyperkinetic movement disorder associated with prolonged dopamine D2 receptor blockade, particularly during chronic haloperidol (HP) exposure. Emerging evidence suggests that TD-like pathology is sustained by an interconnected redox–mitochondrial–inflammatory network within striatal circuits; however, the regulatory architecture of this network remains incompletely defined. Hyperoside (HS), a flavonol glycoside with cytoprotective properties, has been implicated in cellular stress-response modulation, yet its role in antipsychotic-induced motor dysfunction remains unclear. In this study, a six-group mechanistic design was employed in which rats received HP (1 mg/kg, i.p., 21 days) to induce TD-like orofacial dyskinesia (OD), quantified by vacuous chewing movements (VCMs) and tongue protrusions (TPs). HS (30 mg/kg, i.p.) was administered alone or in combination with HP, with or without pharmacological inhibition of nuclear factor erythroid 2–related factor 2 (Nrf2) using ML385. HP exposure induced progressive dyskinetic behavior accompanied by oxidative and nitrosative stress, mitochondrial dysfunction, increased pro-inflammatory cytokines, and elevated caspase-3 activity in the striatum. HS significantly attenuated behavioral abnormalities while restoring redox balance, preserving mitochondrial enzyme activities, and reducing inflammatory and apoptotic signaling. Notably, Nrf2 inhibition intensified molecular pathology without proportionally worsening behavioral outcomes, indicating a dissociation between biochemical vulnerability and overt motor expression. Furthermore, ML385 markedly attenuated HS-mediated protection across multiple endpoints. Collectively, these findings support a potential protective role for Nrf2-related regulatory mechanisms in limiting network destabilization in TD-like pathology, while highlighting the importance of integrated stress-response pathways in modulating disease progression.

## 1. Introduction

Tardive dyskinesia (TD) is a persistent hyperkinetic movement disorder that develops after prolonged exposure to dopamine D2 receptor–blocking agents, particularly typical antipsychotics such as haloperidol (HP). Clinically, TD manifests as repetitive involuntary orofacial movements that significantly impair quality of life and complicate long-term psychiatric management. Although vesicular monoamine transporter 2 (VMAT2) inhibitors provide symptomatic relief, they do not address the molecular processes that sustain neuronal vulnerability and circuit instability [1,2,3]. Accordingly, increasing attention has shifted toward delineating stress-related mechanisms underlying TD development and persistence. Recent mechanistic syntheses indicate that TD pathophysiology extends beyond dopamine receptor supersensitivity and involves progressive destabilization of interconnected redox, mitochondrial, and inflammatory processes within basal ganglia circuitry [4,5,6,7]. Sustained D2 receptor blockade enhances dopamine turnover and oxidative metabolism, increasing reactive oxygen species (ROS) generation in metabolically active striatal neurons. Excessive ROS production promotes lipid peroxidation, glutathione depletion, and impairment of mitochondrial respiratory efficiency, thereby reducing bioenergetic capacity and compromising neuronal resilience. Importantly, oxidative stress and mitochondrial dysfunction reinforce one another through a bidirectional amplification loop, while redox imbalance concurrently activates pro-inflammatory signaling cascades, including TNF-α, IL-1β, and IL-6 release [5,6,8]. This redox–mitochondrial–inflammatory feed-forward network is increasingly recognized as a central pathogenic axis in TD and related movement disorders.

Within this framework, nuclear factor erythroid 2–related factor 2 (Nrf2) has emerged as a key transcriptional regulator of cytoprotective programs. Nrf2 activation induces coordinated expression of antioxidant enzymes, glutathione biosynthesis pathways, detoxification systems, and mitochondrial-supportive genes, thereby integrating multi-layered cellular defense mechanisms. Impaired Nrf2 signaling has been implicated in neurodegenerative and motor disorders characterized by oxidative–inflammatory imbalance [9,10,11]. Nevertheless, the hierarchical role of Nrf2 in orchestrating redox–mitochondrial–inflammatory coupling under antipsychotic-induced TD-like conditions remains insufficiently defined.

Hyperoside (quercetin-3-O-galactoside; HS) is a naturally occurring flavonol glycoside with documented antioxidant, anti-inflammatory, and mitochondrial-supportive properties [12,13,14,15]. Beyond direct radical-scavenging activity, HS has been reported to modulate neuroimmune–redox interactions through engagement of regulatory pathways such as PI3K/AKT and Nrf2/HO-1, thereby contributing to stabilization of oxidative–inflammatory crosstalk and mitochondrial homeostasis [16,17]. However, whether HS mitigates HP-induced TD-like motor dysfunction—and whether such protection depends on Nrf2-mediated regulatory control—remains unclear.

Clarifying whether Nrf2 functions as an upstream homeostatic regulator or merely as a parallel antioxidant pathway requires experimental designs capable of resolving pathway dependency. In particular, it remains unknown whether pharmacological inhibition of Nrf2 directly exacerbates dyskinetic output or instead increases molecular vulnerability without proportionally intensifying behavioral expression. Therefore, the present study employed a six-group mechanistic design incorporating a 21-day HP regimen, HS intervention, and pharmacological Nrf2 inhibition using ML385. By integrating longitudinal behavioral assessment with comprehensive analysis of oxidative/nitrosative stress markers, mitochondrial bioenergetics, inflammatory cytokines, and apoptotic signaling in the striatum, we aimed to (i) define the hierarchical relationships among redox imbalance, mitochondrial dysfunction, and inflammatory activation in TD-like pathology; (ii) determine whether HS stabilizes this interconnected stress network; and (iii) establish the extent to which Nrf2 signaling governs HS-mediated neuroprotection.

## 2. Materials and Methods

### 2.1. Animals

All procedures were conducted in accordance with the Guide for the Care and Use of Laboratory Animals issued by the U.S. National Institutes of Health and were approved by the Institutional Animal Care and Use Committee of the National Taiwan University College of Medicine (IACUC approval no. 20220527). Adult Wistar rats (270–300 g; approximately 3 months old) were obtained from BioLASCO Taiwan Co., Ltd. (Taipei, Taiwan). Animals were housed three per cage in standard Plexiglas cages under controlled environmental conditions (22 ± 3 °C; 12 h light/dark cycle, lights on at 07:00) with free access to food and water. Animal health and body weight were monitored daily. To minimize stress-related variability, rats were habituated to gentle handling (20 min/day) for seven consecutive days prior to experimental procedures. Animals were randomly allocated to treatment groups. At study completion, euthanasia was performed by CO_2_ inhalation in accordance with institutional guidelines.

### 2.2. Drugs

Haloperidol (HP; CAS 52-86-8), ML385 (CAS 846557-71-9), and hyperoside (HS; ≥95% purity; CAS 482-36-0) were purchased from Sigma-Aldrich (St. Louis, MO, USA). HP was dissolved in sterile saline. ML385 and HS were initially dissolved in dimethyl sulfoxide (DMSO) and further diluted with saline to the desired concentrations. The final DMSO concentration did not exceed 1% (*v*/*v*), and corresponding vehicle controls (1% DMSO in saline) were administered to matched groups. All solutions were freshly prepared under sterile conditions prior to use.

Doses were selected based on previous studies [6,8,16,18] and administered intraperitoneally (i.p.) once daily for 21 consecutive days at a fixed volume of 2.0 mL/kg body weight. A preliminary dose-ranging assessment (1–100 mg/kg, i.p.) was performed to evaluate tolerability and behavioral effects in the TD-like paradigm. Based on these observations, 30 mg/kg HS was selected for subsequent experiments, as it produced consistent protective effects without observable adverse outcomes. The intraperitoneal route was selected to minimize pharmacokinetic variability and ensure stable systemic exposure throughout the 21-day regimen.

### 2.3. Experimental Groups and Drug Treatment

Rats were randomly assigned to six experimental groups (*n* = 8 per group). Both male and female animals were included and evenly distributed across groups; sex was not incorporated as a statistical factor due to sample size considerations.

The groups were defined as follows:

1. Vehicle control (CT): vehicle (i.p.) once daily for 21 days
2. HP: HP (1 mg/kg, i.p.) once daily for 21 days
3. HS: HS (30 mg/kg, i.p.) once daily for 21 days
4. HP + HS: HP (1 mg/kg, i.p.) followed 60 min later by HS (30 mg/kg, i.p.), once daily
5. HP + ML: HP (1 mg/kg, i.p.) followed 30 min later by ML385 (30 mg/kg, i.p.), once daily
6. HP + ML + HS: HP (1 mg/kg, i.p.), ML385 (30 mg/kg, i.p.) administered 30 min after HP, followed by HS (30 mg/kg) 30 min later (i.e., 60 min after HP), once daily

**Figure 1:**
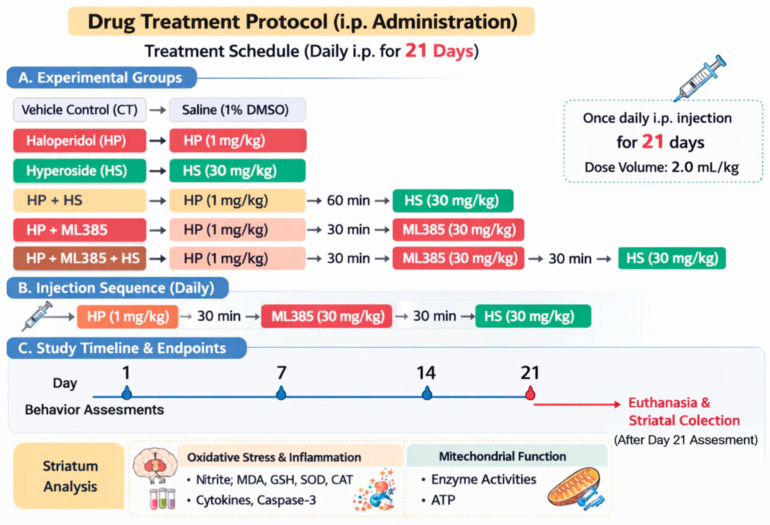
Experimental design and mechanistic overview.

All injections were performed at a consistent time each day. The injection volume was maintained at 2.0 mL/kg body weight. A schematic overview of the treatment timeline is presented in Figure 1.

Rats were assigned to six groups (CT, HP, HS, HP + HS, HP + ML, and HP + ML + HS) and treated once daily for 21 days. HP (1 mg/kg, i.p.) was administered once daily to induce TD-like OD; HS (30 mg/kg, i.p.) was given 60 min after HP, and ML385 (30 mg/kg, i.p.) 30 min before HS. Behavioral testing was performed on days 1, 7, 14, and 21, followed by striatal collection on day 21. The scheme illustrates the experimental strategy used to assess the potential involvement of Nrf2-related regulation in redox, mitochondrial, and inflammatory dysfunction.

### 2.4. Behavioral Assessment of OD

OD was quantified as previously described [6,8,18]. Behavioral assessments were performed 6 h after HP or vehicle administration on experimental days 1, 7, 14, and 21. Rats were individually placed in a transparent observation chamber (20 × 20 × 19 cm) equipped with angled mirrors beneath the floor to allow unobstructed visualization of orofacial movements. Following a 2 min habituation period, vacuous chewing movements (VCMs) and tongue protrusions (TPs) were recorded for 5 min. VCMs were defined as single vertical jaw movements not directed toward physical material, whereas TPs were defined as protrusions of the tongue beyond the incisors. To minimize observer bias, animals were randomly coded prior to testing, and behavioral scoring was performed independently by two investigators blinded to treatment allocation. All behavioral assessments were conducted between 09:00 and 11:00 a.m. under consistent environmental conditions.

### 2.5. Biochemical Measurements

On day 21, animals were euthanized after the final behavioral assessment. Brains were rapidly removed, rinsed in ice-cold isotonic saline, and the striatum was dissected on an ice-cold surface according to a standard stereotaxic atlas [19]. Tissue samples were immediately processed for biochemical analyses. For oxidative stress and inflammatory assays, striatal tissue was homogenized (10%, *w*/*v*) in ice-cold 0.1 M phosphate buffer (pH 7.4) using a glass–Teflon homogenizer (Schuett-biotec GmbH, Göttingen, Germany) at 4 °C. Homogenates were centrifuged at 1000× *g* for 10 min at 4 °C, and the resulting post-nuclear supernatant was used for measurement of nitrite, MDA, GSH, SOD, CAT, cytokines, and caspase-3 activity unless otherwise indicated. For mitochondrial assays, a separate portion of striatal tissue was homogenized in ice-cold mitochondrial isolation buffer (0.25 M sucrose, 10 mM Tris-HCl, pH 7.4), centrifuged at 1000× *g* for 10 min to remove debris, and then centrifuged at 10,000× *g* for 20 min at 4 °C to obtain the crude mitochondrial pellet. The pellet was washed and resuspended in mannitol–sucrose–HEPES buffer (pH 7.4) for enzymatic analyses. Protein concentrations in cytosolic and mitochondrial fractions were determined using the Lowry method [20] with bovine serum albumin as the standard.

### 2.6. Oxidative and Nitrosative Stress Markers

Nitrite concentrations were quantified using the Griess reaction at 540 nm [21] and calculated from a sodium nitrite standard curve (6–600 μM). Malondialdehyde (MDA) levels were measured using the thiobarbituric acid reactive substances (TBARS) assay at 532 nm [22] and expressed as nmol/mg protein. Reduced glutathione (GSH) content was measured using Ellman’s reagent at 412 nm [23] and expressed as nmol/mg tissue. Superoxide dismutase (SOD) and catalase (CAT) activities were determined spectrophotometrically according to established methods [24,25]. SOD activity was normalized to total protein and expressed as units/mg tissue. CAT activity was expressed as units/mg tissue.

### 2.7. Mitochondrial Function

Mitochondrial bioenergetic status was evaluated by measuring the activities of succinate dehydrogenase (SDH), NADH–cytochrome C reductase (complex I–III), succinate–cytochrome C reductase (complex II–III), and total ATPase activity. SDH activity was determined using the p-iodonitrotetrazolium violet reduction assay [26]. Mitochondrial protein (0.05 mg) was incubated in 50 mM potassium phosphate buffer (pH 7.4) containing 10 mM sodium succinate and 2.5 μg/mL p-iodonitrotetrazolium violet. After incubation, the reaction was terminated with 10% trichloroacetic acid, and absorbance was measured at 490 nm. Total ATPase activity was quantified by measuring inorganic phosphate released from ATP [27]. Reactions were performed at 37 °C and terminated with 20% trichloroacetic acid. Inorganic phosphate was measured colorimetrically and expressed as μg Pi/mg protein. Complex I–III and complex II–III activities were determined spectrophotometrically according to Navarro et al. [28]. All mitochondrial enzyme activities were normalized to mitochondrial protein content.

### 2.8. Neuroinflammatory and Apoptotic Markers

Striatal TNF-α, IL-1β, and IL-6 levels were measured using commercially available ELISA kits (KRISHGEN BioSystem, Whittier, CA, USA) according to the manufacturer’s instructions. Cytokine concentrations were calculated from standard curves and expressed as pg/mg protein. Caspase-3 activity was determined using a colorimetric assay based on p-nitroaniline (pNA) cleavage and expressed as nmol pNA released per mg protein.

### 2.9. Statistical Analysis

All data are expressed as mean ± standard error of the mean (SEM). The normality of data distribution was evaluated using the Shapiro–Wilk test prior to selecting parametric statistical methods. Behavioral outcomes, including vacuous chewing movements (VCMs) and tongue protrusions (TPs), were analyzed using a two-way repeated-measures ANOVA, with treatment as the between-subject factor and time as the within-subject factor. Where appropriate, significant effects were further examined using Tukey’s post hoc multiple comparison test. For repeated-measures analyses, the assumption of sphericity was assessed using Mauchly’s test, and Greenhouse–Geisser correction was applied when violations were detected. Biochemical data were analyzed using one-way ANOVA followed by Tukey’s post hoc test for multiple comparisons. To provide an estimate of the magnitude of treatment effects, partial eta squared (partial η^2^) values were calculated for all ANOVA models. Exact *p* values are reported unless they were below 0.001. Statistical analyses were conducted using GraphPad Prism software (version 8.3.0; GraphPad Software, San Diego, CA, USA). A *p* value < 0.05 was considered statistically significant. No data transformation or rescaling was applied prior to statistical analysis.

## 3. Results

### 3.1. HS Attenuates HP-Induced TD-Like OD

**Figure 2:**
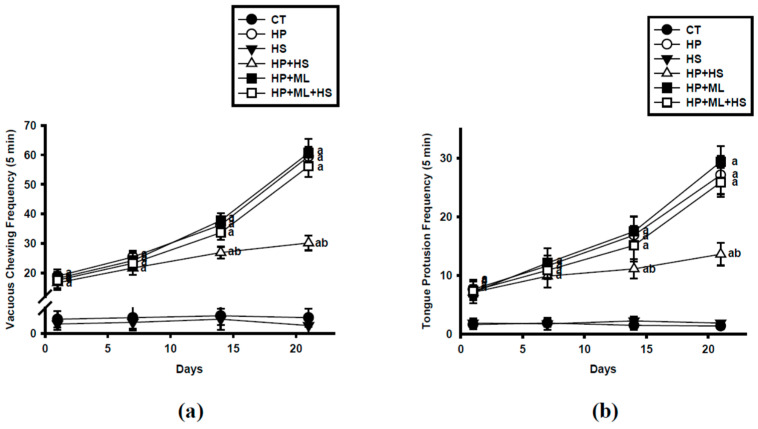
HS Attenuates Progressive OD Behavior. (**a**) Vacuous chewing movements (VCMs) and (**b**) tongue protrusions (TPs) over the 21-day treatment period. HP progressively increased OD behaviors, whereas HS significantly attenuated these changes, particularly at later time points. ML385 did not further exacerbate dyskinesia but markedly attenuated HS-mediated behavioral protection. Data are presented as mean ± SEM (*n* = 8 per group). Repeated-measures two-way ANOVA followed by Tukey’s post hoc test was used for statistical analysis, with Greenhouse–Geisser correction applied when appropriate. “a” *p* < 0.001 vs. CT; “b” *p* < 0.001 vs. HP.

Repeated-measures two-way ANOVA revealed a significant main effect of treatment on VCMs (Figure 2a) (F(5,42) = 1548.72, *p* < 0.001, partial η^2^ = 0.995), a significant main effect of time (F(1.12, 47.04) = 902.36, *p* < 0.001, partial η^2^ = 0.957; Greenhouse–Geisser corrected), and a significant treatment × time interaction (F(15,126) = 118.94, *p* < 0.001, partial η^2^ = 0.934). Mauchly’s test indicated that the assumption of sphericity was violated (W = 0.0152, *p* < 0.001); therefore, Greenhouse–Geisser correction was applied. Similar effects were observed for TPs (Figure 2b) (treatment: F(5,42) = 1426.58, *p* < 0.001, partial η^2^ = 0.994; time: F(1.15,48.21) = 812.47, *p* < 0.001, partial η^2^ = 0.951; interaction: F(15,126) = 109.36, *p* < 0.001, partial η^2^ = 0.929). Post hoc comparisons demonstrated that HP-treated rats exhibited progressively increased VCMs and TPs compared with CT, reaching statistical significance on days 14 and 21 (*p* < 0.001 vs. CT), confirming progressive induction of TD-like OD. The large F-values reflect low within-group variability and strong group separation.

HS administered alone did not significantly alter OD behaviors relative to CT (all *p* > 0.05), indicating absence of nonspecific motor effects. Co-treatment with HS significantly attenuated HP-induced VCMs and TPs (*p* < 0.001 vs. HP), particularly at later time points, demonstrating sustained protective efficacy. ML385 did not significantly exacerbate HP-induced OD behaviors (HP + ML vs. HP; *p* > 0.05). However, ML385 markedly attenuated HS-mediated behavioral protection, as VCMs and TPs in the HP + ML + HS group were not significantly different from the HP group (*p* > 0.05) and remained significantly higher than those in the HP + HS group (*p* < 0.001).

### 3.2. HS Restores HP-Induced Redox Imbalance

**Figure 3:**
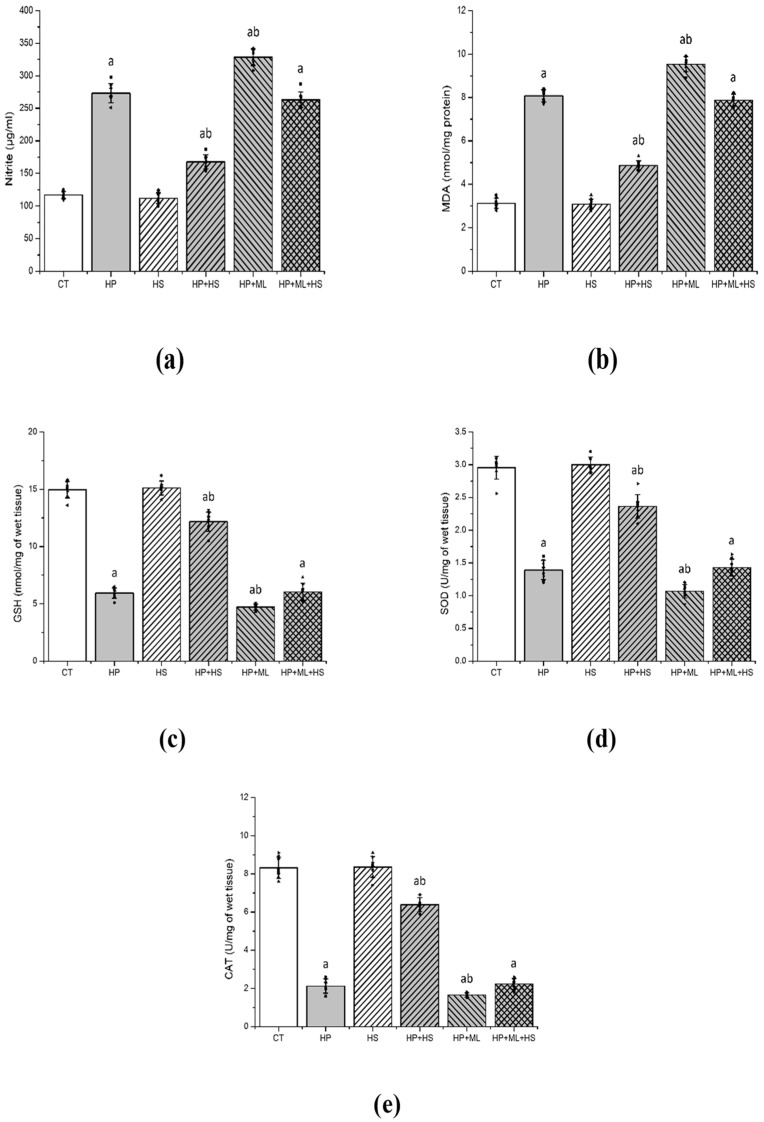
HS restores redox homeostasis. Striatal oxidative and nitrosative stress markers: (**a**) nitrite, (**b**) MDA, (**c**) GSH, (**d**) SOD, and (**e**) CAT. HP induced marked redox imbalance, whereas HS restored antioxidant defenses and reduced oxidative damage. ML385 further aggravated oxidative stress and markedly attenuated HS-mediated redox restoration. Data are presented as mean ± SEM with individual data points shown (*n* = 8 per group). One-way ANOVA followed by Tukey’s post hoc test. “a” *p* < 0.001 vs. CT; “b” *p* < 0.001 vs. HP.

One-way ANOVA revealed significant group effects for nitrite (Figure 3a) (F(5,42) = 302.18, *p* < 0.001, partial η^2^ = 0.973), MDA (Figure 3b) (F(5,42) = 412.76, *p* < 0.001, partial η^2^ = 0.98), GSH (Figure 3c) (F(5,42) = 512.87, *p* < 0.001, partial η^2^ = 0.984), SOD activity (Figure 3d) (F(5,42) = 589.44, *p* < 0.001, partial η^2^ = 0.986), and CAT activity (Figure 3e) (F(5,42) = 468.95, *p* < 0.001, partial η^2^ = 0.982). HP significantly increased nitrite and MDA levels and reduced GSH content and antioxidant enzyme activities compared with CT (*p* < 0.001).

ML385 significantly intensified HP-induced oxidative stress: the HP + ML group exhibited further elevations in nitrite and MDA and additional reductions in GSH, SOD, and CAT compared with HP alone (*p* < 0.001), indicating further redox destabilization under Nrf2 inhibition. HS co-treatment significantly restored redox parameters (*p* < 0.001 vs. HP), whereas ML385 attenuated these protective effects. Values in the HP + ML + HS group were not significantly different from those in the HP group for key oxidative markers (*p* > 0.05), indicating loss of HS-mediated redox stabilization under Nrf2 blockade.

### 3.3. HS Preserves HP-Induced Mitochondrial Dysfunction

**Figure 4:**
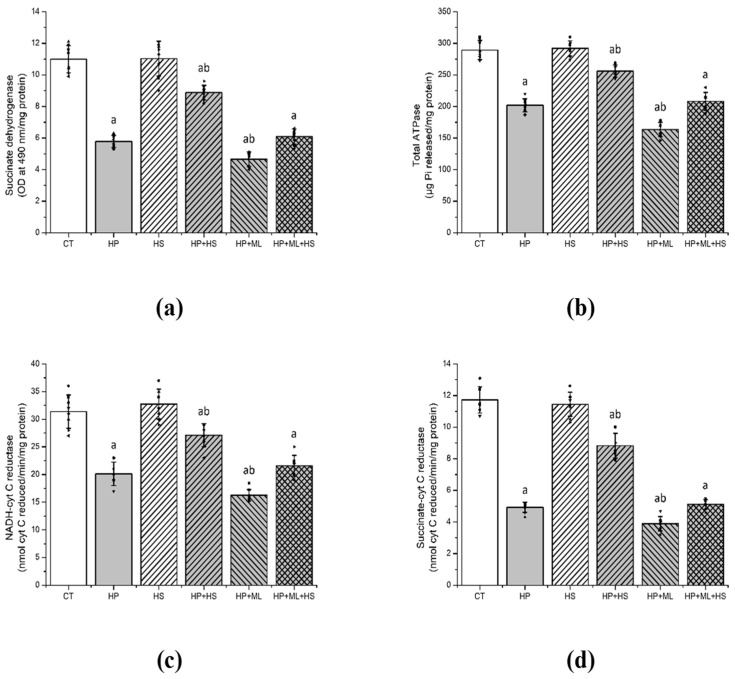
HS preserves mitochondrial bioenergetic function. Mitochondrial parameters in the striatum: (**a**) SDH, (**b**) total ATPase activity, (**c**) NADH–cytochrome C reductase activity, and (**d**) succinate–cytochrome C reductase activity. HP significantly impaired mitochondrial enzyme activities, consistent with bioenergetic dysfunction. HS significantly preserved mitochondrial function, whereas ML385 further aggravated mitochondrial impairment and markedly attenuated HS-mediated protection. Data are presented as mean ± SEM with individual data points shown (*n* = 8 per group). One-way ANOVA followed by Tukey’s post hoc test. “a” *p* < 0.001 vs. CT; “b” *p* < 0.001 vs. HP.

Significant group effects were observed for SDH activity (Figure 4a) (F(5,42) = 329.54, *p* < 0.001, partial η^2^ = 0.975), ATPase activity (Figure 4b) (F(5,42) = 177.64, *p* < 0.001, partial η^2^ = 0.955), NADH–cytochrome C reductase activity (Figure 4c) (F(5,42) = 128.94, *p* < 0.001, partial η^2^ = 0.939), and succinate–cytochrome C reductase activity (Figure 4d) (F(5,42) = 402.68, *p* < 0.001, partial η^2^ = 0.98). HP significantly impaired mitochondrial enzyme activities relative to CT (*p* < 0.001). The HP + ML group exhibited further reductions in mitochondrial function compared with HP alone (*p* < 0.001), indicating increased mitochondrial vulnerability under Nrf2 inhibition.

Despite this molecular exacerbation, ML385 did not produce additional worsening of OD behaviors (Section 3.1), indicating a dissociation between biochemical network destabilization and overt motor expression. HS significantly preserved mitochondrial enzyme activities compared with HP (*p* < 0.01), whereas ML385 attenuated this mitochondrial protection in the HP + ML + HS group.

### 3.4. HS Suppresses HP-Induced Neuroinflammatory Responses

**Figure 5:**
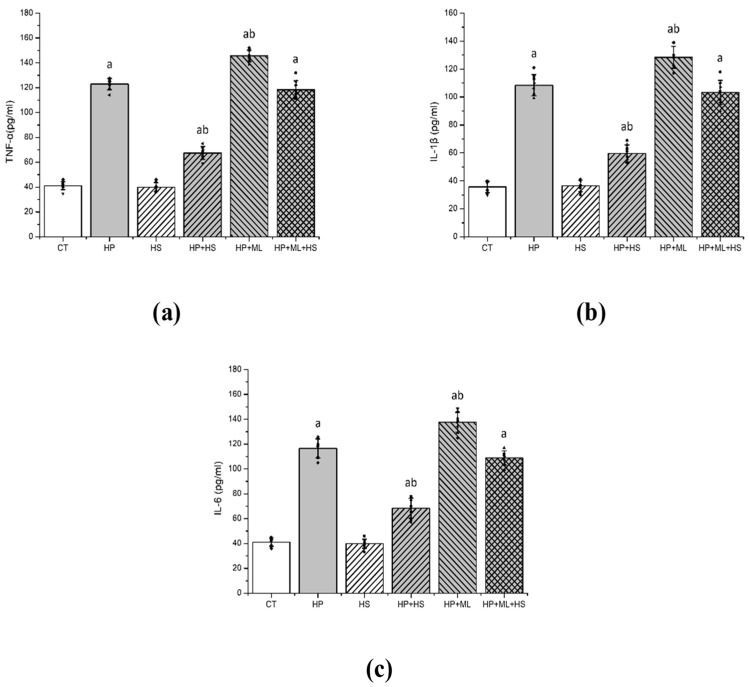
HS suppresses neuroinflammatory responses. Striatal levels of (**a**) TNF-α, (**b**) IL-1β, and (**c**) IL-6. HP induced robust neuroinflammatory activation, whereas HS significantly reduced cytokine expression. ML385 further amplified inflammatory signaling and markedly attenuated HS-mediated protection, consistent with a role for Nrf2 in restraining inflammatory propagation. Data are presented as mean ± SEM with individual data points shown (*n* = 8 per group). One-way ANOVA followed by Tukey’s post hoc test. “a” *p* < 0.001 vs. CT; “b” *p* < 0.001 vs. HP.

One-way ANOVA revealed significant group effects for TNF-α (Figure 5a) (F(5,42) = 621.84, *p* < 0.001, partial η^2^ = 0.987), IL-1β (Figure 5b) (F(5,42) = 403.72, *p* < 0.001, partial η^2^ = 0.98), and IL-6 (Figure 5c) (F(5,42) = 458.27, *p* < 0.001, partial η^2^ = 0.983). HP significantly elevated cytokine levels compared with CT (*p* < 0.001). ML385 further increased TNF-α, IL-1β, and IL-6 relative to HP alone (*p* < 0.001), consistent with amplification of inflammatory signaling under Nrf2 inhibition. HS significantly suppressed cytokine production (*p* < 0.01 vs. HP), whereas ML385 attenuated HS-mediated anti-inflammatory effects.

### 3.5. HS Attenuates HP-Induced Apoptotic Activation

**Figure 6:**
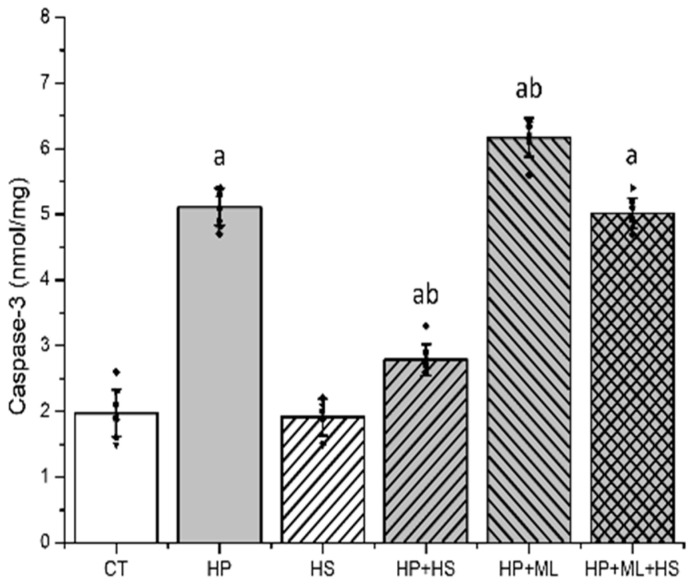
HS attenuates apoptotic signaling. Striatal caspase-3 activity. HP increased apoptotic signaling, consistent with downstream convergence of oxidative, mitochondrial, and inflammatory stress. HS significantly reduced caspase-3 activation, whereas ML385 enhanced apoptotic activity and markedly attenuated HS-mediated protection. Data are presented as mean ± SEM with individual data points shown (*n* = 8 per group). One-way ANOVA followed by Tukey’s post hoc test. “a” *p* < 0.001 vs. CT; “b” *p* < 0.001 vs. HP.

Caspase-3 activity differed significantly among groups (Figure 6) (F(5,42) = 336.29, *p* < 0.001, partial η^2^ = 0.976). HP significantly increased caspase-3 activity compared with CT (*p* < 0.001). ML385 further elevated apoptotic signaling relative to HP alone (*p* < 0.001). HS significantly reduced caspase-3 activation (*p* < 0.001 vs. HP), whereas ML385 attenuated this protective effect (*p* = 0.983), consistent with enhanced convergence of oxidative and inflammatory stress on apoptotic signaling under Nrf2 inhibition. No significant difference was observed between CT and HS groups (*p* = 0.998).

## 4. Discussion

The present six-group mechanistic investigation demonstrates that HS attenuates HP-induced TD-like OD through coordinated stabilization of redox homeostasis, mitochondrial bioenergetics, neuroinflammatory signaling, and apoptotic cascades within the striatum. Repeated HP administration for 21 consecutive days produced progressive increases in VCMs and TPs, confirming the translational validity of this model. HS alone did not induce motor abnormalities yet significantly reduced HP-evoked dyskinesia, supporting a sustained neuroprotective effect rather than nonspecific motor suppression.

A central observation is the close association between behavioral improvement and restoration of striatal redox equilibrium. HP exposure produced marked oxidative and nitrosative stress, reflected by increased malondialdehyde and nitrite levels, depletion of reduced glutathione, and suppression of SOD and CAT activities. These findings are consistent with contemporary models identifying oxidative stress as a major contributor to TD pathophysiology [4,6,7,8]. Sustained D2 receptor blockade enhances dopamine turnover and oxidative metabolism, thereby increasing ROS generation and destabilizing redox homeostasis [6]. Oxidative stress and mitochondrial dysfunction form a bidirectional amplification loop, in which impaired respiratory chain activity further increases ROS production, reinforcing cellular instability [29,30]. Within this framework, HS restored both enzymatic and non-enzymatic antioxidant defenses, suggesting interruption of feed-forward oxidative amplification loops that perpetuate neuronal vulnerability under prolonged neuroleptic exposure [6,14,15,16]. The magnitude and consistency of normalization across multiple redox indices support the interpretation that oxidative destabilization functions as an early and central contributor within the broader pathological network [6,30].

Inclusion of the HP + ML and HP + ML + HS groups enabled direct interrogation of Nrf2 pathway dependency. Pharmacological inhibition of Nrf2 aggravated oxidative stress, mitochondrial impairment, inflammatory activation, and caspase-3 activity in HP-treated animals, yet did not proportionally exacerbate OD behaviors. This dissociation suggests a threshold relationship between biochemical destabilization and overt motor expression. These findings suggest a systems-level regulatory role for Nrf2 that constrains oxidative–inflammatory escalation rather than directly modulating motor output [9,10]. Loss of Nrf2-mediated regulation increased molecular vulnerability and intensified network destabilization without proportionally amplifying dyskinetic expression, indicating that biochemical stress burden and motor manifestation are not linearly coupled [5,8,9,10].

Importantly, Nrf2 inhibition markedly attenuated HS-mediated protection across both behavioral and molecular endpoints, supporting the interpretation that Nrf2-dependent regulatory control is a major mediator of HS-induced neuroprotection. However, a partial contribution of Nrf2-independent mechanisms cannot be entirely excluded, and the present findings therefore suggest that Nrf2 signaling is a principal, but potentially not exclusive, pathway underlying the protective effects of HS [9,10,15,16]. Because direct measurements of Nrf2 activation were not performed in the present study, these conclusions should be interpreted as pharmacological evidence of pathway involvement rather than definitive proof of Nrf2 activation.

Mitochondrial dysfunction emerged as a downstream consequence of redox destabilization [30,31]. HP significantly reduced SDH, complex I–III, complex II–III, and ATPase activities, indicating compromised oxidative phosphorylation and impaired bioenergetic capacity [5,6,8]. Because mitochondria both generate and are damaged by ROS, redox–mitochondrial coupling establishes a self-reinforcing pathological loop under sustained dopaminergic stress [29,30].

HS preserved mitochondrial enzyme activities and bioenergetic indices, suggesting that stabilization of redox balance mitigates secondary mitochondrial collapse [6,16,30]. Conversely, ML further reduced mitochondrial function, reinforcing the role of Nrf2 in maintaining mitochondrial integrity [8,31]. The absence of proportional behavioral worsening despite aggravated mitochondrial dysfunction supports a threshold model in which bioenergetic compromise primarily influences long-term neuronal resilience rather than immediate motor manifestation [6,30].

HP administration elevated TNF-α, IL-1β, and IL-6 levels in the striatum, consistent with immune–oxidative models of TD and extrapyramidal syndromes [4,5,8]. Pro-inflammatory cytokines potentiate oxidative stress via NADPH oxidase activation and mitochondrial disruption, while oxidative injury enhances inflammatory transcriptional programs, forming a bidirectional amplification cycle [30,32].

HS suppressed inflammatory mediators, whereas ML amplified cytokine expression in HP-treated animals, indicating that Nrf2-dependent regulation restrains inflammatory escalation within the interconnected stress network [5,16]. Parallel modulation of caspase-3 activity indicates convergence of oxidative and inflammatory destabilization on apoptotic pathways [5,8]. Together, these findings support a hierarchical architecture in which Nrf2 appears to function as an upstream regulatory contributor, mitochondrial preservation, inflammatory restraint, and apoptotic limitation [16,30,32].

While the HP-induced OD model reproduces several key behavioral and molecular features relevant to TD, it does not fully capture the clinical heterogeneity or long-term disease course observed in patients [1,2,4,6]. Although both male and female animals were included, the study was not designed or powered to detect sex-specific effects; accordingly, these findings should be interpreted with caution in this regard. Reliance on pharmacological inhibition also limits pathway specificity, and future studies incorporating genetic or conditional Nrf2 approaches would strengthen mechanistic resolution. In addition, other processes, including synaptic plasticity alterations and corticostriatal circuit remodeling, may contribute to TD-like pathology and warrant further investigation. Finally, because HS was administered intraperitoneally in this preclinical setting, the dosing paradigm may not directly reflect clinically relevant exposure conditions, and further pharmacokinetic and translational studies will be required to define its therapeutic potential in humans.

## 5. Conclusions

In conclusion, HS mitigates HP-induced TD-like pathology by modulating interconnected redox, mitochondrial, inflammatory, and apoptotic processes within the striatum. The attenuation of HS-mediated protective effects following pharmacological inhibition of Nrf2 suggests that Nrf2-related pathways contribute to maintaining network stability under conditions of chronic dopaminergic disruption. However, as direct measures of Nrf2 activation were not included, these findings should be interpreted as evidence of pathway involvement rather than definitive proof of Nrf2 activation. The observed dissociation between molecular alterations and behavioral outcomes further supports a threshold-based model of TD-like pathology, in which substantial biochemical disruption does not necessarily translate linearly into motor expression. Taken together, this study highlights the potential relevance of targeting integrated stress-response networks, rather than single downstream pathways, as a strategy for modulating TD-like disease processes.

## Data Availability

The datasets generated and analyzed during the current study are available from the corresponding author upon reasonable request. Please note that these data are not publicly accessible in order to maintain confidentiality and protect participant privacy.

## Abbreviations

The following abbreviations are used in this manuscript:

CAT	Catalase
GSH	Reduced glutathione
HP	Haloperidol
HS	Hyperoside
MDA	Malondialdehyde
Nrf2	Nuclear factor erythroid 2–related factor 2
OD	Orofacial dyskinesia
ROS	Reactive oxygen species
SDH	Succinate dehydrogenase
SOD	Superoxide dismutase
TBARS	Thiobarbituric acid reactive substances
TD	Tardive dyskinesia
TPs	Tongue protrusions
VCMs	Vacuous chewing movements
VMAT2	Vesicular monoamine transporter 2
